# General Lymphangitis*Read before the California State Veterinary Association, December 13th, 1888. Reprinted from the American Veterinary Review.

**Published:** 1889-10

**Authors:** J. P. Klench


### Art. XXIII.—GENERAL LYMPHANGITIS.*

By J. P. Klench, V.S.

There has been, for the last six years, existing among the equine species of California, and more especially amongst the mules, a general constitutional disease, which has proved fatal in nearly every instance, and that has caused death after several months of loathsome suffering and of pitiful distress.

This affection has not been, to my knowledge, submitted to a diligent study by any practitioner, nor has there been any particular description given of it by the veterinarian authors, with the exception of a few practical records that have appeared in some veterinary publications.

I had the good fortune of following several cases in various localities of two counties from the very first start of the disease to the time the animals were destroyed, and took careful notice of the origin and the progress of the symptoms, as well as of the post-mortem lesions. I am glad to have the opportunity to lay my opinion before a meeting of veterinary surgeons for their critical perusal.

*Causes.*—Up to five or six years ago, this affection was little known, and has perhaps never been noticed before. Long continued rains and a subsequent inundation of the lower lands in all the valleys, caused the atmosphere to be moist, the nights chilly with heavy fogs that seldom cleared away before noon. Then it was that five or six months after that flood, this disease made its appearance amongst the mules of Roberts Island, near Stockton,

*Read before the California State Veterinary Association, December 13th, 1888. Reprinted from the American Veterinary Review.

and all along the tules and rivers, but soon the affection spread over the whole San Joaquin valley, up to the foothills, so that hardly any ranch in the valley was spared. Work, care or feed cannot be accused of having any influence in the development of this disease, as animals have been observed to be attacked while in pasture or at rest in the stable and adjoining corrals, receiving at the time a very substantial nourishment.

The question of contagion might be considered as causative, but must be rejected as doubtful, until evident proofs are brought forward based on direct inoculation. My own experience is rather a proof against the contagious character of the affection, as I will relate. In the majority of cases, the disease was in all certainty of a spontaneous origin, and could not be traced to any particular cause. It seems rather to be due to a special climatic influence, having the same effect in distant localities of the State at the same time.

Physiological works tell us that the secreting organs or glands are far less active in mules than in horses, and daily experience teaches us that in general, mules will perspire and urinate less than their equine companions in the same team. A similar fact has been noticed concerning the salivary and intestinal glands. This circumstance explains why mules require less water for daily use, and why their muscles and tissues are more dense, more hard, more tenacious and resisting. They also tell us why the blood of the mule is less deprived of its impure deleterious elements, and, therefore induces me to consider this impure condition of the blood as a *possible* cause of this disease.

*Symptoms.*—They are regular in their expression and have but two modes of eruption. The disease might break out on the legs and the body, or in the upper passages of the respiratory organs. The first indication of the disease will be dullness, loss of appetite and falling off in general condition, which might take place two or three weeks before any other symptoms appear. Swellings will then be noticed on one or several legs, causing lameness, or on any other part of the body; sometimes they affect the upper parts of the limbs more than the lower parts, and generally send out one or several cords to the nearest ganglion. They are painful, more or less œdematous, of firm, but not hard resistance, and will be covered in a few days with numerous lymphatic cords, especially when they are located over the muscular regions of the hind legs, neck and breast. In a few days more these cords, soft on pressure, become knotty ; every lump becomes soft, opens and forms a wound, giving exit to yellow-whitish fluid, resembling thick coagulated lymph. Those abscesses located on the lymphatic vessels, evacuate a great amount of fluid, coming from two, four or six more buttons, situated on the same vessel. After the opening of the abscesses, the lymphatic vessels retract, remain soft so that they can hardly be felt. Such buttons as are located on hard underlying tissues, like joints or bones, are small, and situated in the derm of the skin, containing only a little fluid. The tumors on the muscles are located deep, cause a great deal of pain when the animal contracts the muscles, and take eight to ten days to reach the skin. They are large and form a regular cavity. It is noticeable that when a great many abscesses have opened, the animal shows relief, his appetite improves and his movements are easier.

The sores resulting from these abscesses are red, rough, bleeding easily, with smooth borders injected and swollen, but not indurated, and secreting healthy matter. Most of the sores will heal and never conglomerate. The smaller sores will cover with a brown sticking crust, upon the removal of which I never noticed the wound to have the tendency to become larger in circumference or deeper at the bottom. The large abscesses on the muscles will, after being opened with the bistoury, often close and fill up again. The fluid running out of the abscesses in vessels always retains the same character, unless farcy complications arrive, as will be shown later. These wounds generally take on a healing process, and many wlll cicatrize in the first period of the disease, when the animal is yet possessing his natural strength and vitality, while at a later stage general debility reducing the vital powers, retards and obstructs the cicatrizing process. Especially is this the case when the affection narrows the respiratory passages and seriously interferes with the proper ærization of the blood in the lungs.

It has been observed in all cases that in a few days after the appearance of the first abscesses, more lumps will daily form on different parts of the body and pass through the various stages of tumors, abscesses and running sores, so that in a short time the animal is literally covered with cords, lumps, abscesses and suppurating wounds that give the most disgusting sight one could have of a living animal.

But in the meanwhile complications arise in the form of gangrene in the nasal membrane. This symptom very often appears before the outbreak on the skin and constitutes the second modus of eruption of this disease.

At first there will be a light watery discharge for a few days; nasal membrane red, and congested; then a large brown crust will be seen sticking to the nasal septum, caused by the drying up of the matter secreted from the surface of a large wound; nasal discharge heavy, white, foamy, watery, mixed with blood and serosity, of a bad odor or not, falling down without sticking to the borders of the nostrils. Inside of nasal cavity there will be discovered sores of various sizes, from that of a pea to that of two or three fingers, extending far upwards in the cavity; sometimes parcels of the membrane hanging loose, being only partially detached; the cartilage bare, necrosed, thin, of a dirty bluish-green color, and most often pierced through, opposite the nostrils, so as to cause communication between the two cavities. I am convinced, from my observation, that this destruction of cartilage is not due to ulceration, but is caused by gangrene, due to want of nourishment after all the vessels have been destroyed. All these sores are red, injected, with a rough, soft bottom, bleeding easily, with borders congested and tumefied. The membrane of nasal wing is smooth, tumefied, shining and often ecchymotic. Every day the sores are increasing in surface. Sometimes small spots will be noticed, greatly resembling a glandered ulcer, but upon closer examination I could never detect any induration; nostrils are sometimes a little swollen; very seldom does the conjunctive membrane show any modification. Intermaxillary glands are soft, long or very large, painful, irregular, knotty on their surface, two or three glands existing at the same time. All are movable and free of any adherence with bone or skin. Respiration will be very laborious, wheezing on account of œdema glottidis.

During all this time the animals have a shining, lustrous coat, skin loose and hair smooth and glossy. They eat little and grow poorer from day to day, so that at the end of one month or so, if they are allowed to live, they become entirely emaciated. But some keep up a good appetite and remain in good condition for a long time, having a tolerably fair appearance yet after five or six months.

*Post-mortem examination.*—Lungs, liver, spleen and kidneys in a normal condition, perfectly sound. Bronchial ganglion lightly tumefied, two intermaxillary glands swollen, soft, loose, resembling a lymphatic vessel duplicated, full of coagulated lymph. Several buttons in the derm hard, a few of them beginning to soften. Nasal cavity, membrane of both sides rotten, gangrened in toto; lower part of septum pierced; cartilage bare to the width of 1½ inch by 3 inches long; parcels of membrane œdematous, congested and ready to sequestrate; here and there a spot more or less wide, having the appearance of an ulcer; but the borders are red, smooth, irregular, not indurated; fold of nasal wing a little œdematous, but membrane smooth and regular. Inferior turbinated bone full of suppuration and partially gangrened or congested; the superior turbinated bone less affected, only a few spots attacked by gangrenous ulceration. In larynx one spot necrosed on the epiglottis; mucous membrane less vascular, but tumefied; small spots of the size of the head of a pin, like beginning of necrosis. All muscles of the body diminished in volume, of very black color, the intra-muscular connective tissue very dense, while the adipose tissue between the muscular fascioli is totally absent. A great deal of fat was found outside and between the layers of the abdominal muscles; large adipose tissue in the mesentery and around the large intestines as well as on the costal regions. I was surprised to see the intra-muscular absorption so great, while the adipose tissue remained abundant in other parts of the body.

*Diagnosis.*—It is very difficult to form a positive diagnosis of this affection on account of its recent origin and its numerous points of resemblance to other diseases, and above all to glanders and farcy. I cannot deny having found many animals that have been suffering for some time where the benign and the malign type exist together, and then the farcinous character becomes the most prominent symptom.

From the description given in the Text-Books of glanders and farcy and of the differential symptoms, I form the following conclusions as to the possibility of glanders.

1. That the mule disease in California is, originally, not the chronic farcy, on account of the complete absence of induration in all the pathological formations—*no hard, adherent gland, no indurated nasal ulcer, no chronic glanders, no hard, indurated cords and ulcers on the skin, no chronic farcy.*

2. That although there exists a very great analogy of symptoms, it cannot be either the acute glanders or farcy, because the mules resist this affection many months, and the horses even longer, while the confirmed acute glanders causes death in about six days. When farcy causes death, the post-mortem examination will always show the lesions of acute glanders in the nasal cavity and lungs, as also of supperative anthritis, but I never discovered any lesion of metastatic or inflammatory origin in the lungs nor in any other viscera of the several mules I have examined after death.

On the other hand, it is an admitted fact that the acute glanders and farcy select in preference animals of a sanguine constitution, and especially the jackass and mule, stallions and well bred horses, which are naturally more excitable than the phlegmatic, cold blooded horse. It has been repeatedly proved that chronic farcy inoculated from a horse to a jackass, never developed the chronic glanders, but always the acute form, and caused death in a few days. Now the mule has evidently inherited a portion of this natural aptitude to contract the acute form in all internal diseases; and, indeed, mules suffering from a chronic internal affection are very, very scarce. It is well known that thousands of mules were affected in this State and lived for several months, some of them even keeping up a good condition for a long time, so that this circumstance must discard all idea that the violent poison of acute glanders and farcy exists in this affection.

I will now consider the question of contagion which undoubtedly highly characterizes glanders and farcy. The main objections I find against the supposed contagion are: 1st, the fact that many animals are attacked in a very short time on the same premises, and at different places of the same county. 2d, the outbreak of the disease over the whole body in a violent manner (which is only possible in the acute glanders), seems opposed to the idea that the multiplication of the bacillus requires time and slow progress, as is the case in chronic farcy (which disease is not indicated by the existing symptoms). 3d, That no evidence is known of the existence of a real, living poison. 4th, That the cessation of the disease at every place after the affected animals were destroyed, and when no measures of disinfection were taken, proves that the disease was located in the individual organism. 5th, That no reliable evidence of direct contagion has been reported, but rather the proofs of non-contagion. 6th, The fact that, in the majority of cases, this disease is spontaneously developed without any known cause, while the glanders are considered to be the product of contagion.

If this disease is not the glanders, what then is its real nature? It is very easy to ask this question, but rather difficult to give it a decisive answer. I acknowledge that on examination of the first few cases, I was impressed by the great similarity of symptoms between this affection and the acute glanders and farcy, but careful examination and repeated observations before and after death created in my mind a doubt as to the real character, which doubt I cannot expel until I am convinced of an adverse opinion by several evident proofs of contagion, and the existence of a glandered bacillus. The idea of chronic farcy and glanders must be rejected, unless we admit the theory that the peculiar climate of California is not adapted to favor the process of induration in the glands and other tissues, or to effect metastatic deposits in the internal organs. This idea, it appears to me, is, however, not admissible, because it is sufficiently demonstrated that tuberculosis is as common amongst cattle and hogs here as in other States, and that consumption amongst people is as frequently originated in California as elsewhere. Besides, no difference has been noticed for this climate, either in the symptoms or in the metastatic deposits.

I can affirm most positively to have seen and condemned for having the glanders, several horses with all the capital symptoms of the chronic type, having an indurated gland and miliary tubercles. It is also very important to mention the peculiar circumstance, that this disease was never found to break out on those ranches where only horses are raised and kept, especially when these horses were of good breed; while it attacked in every case animals in farms where we find working horses and mules together, or mules only. I have yet to learn of a well bred horse falling a victim to this affection.

To resume, if we conclude to admit this to be the acute glanders and farcy, (or the sub-acute type), we must accept that the glandered poison has lost its intensity and that its effects remain external and never form any metastatic deposits ; that there is no contagion, but only infection ; and finally, that the disease is spontaneously developed and not the products of contagion.

*Ergo.* The old theories, advocated by the greatest veterinary authorities of all nations, about the nature of glanders, are declared false and without foundation—for California.

I am convinced that the affection is located in the lymphatic system, and consists either in a certain modification of the lymphatic fluid or in an inflammation of the vessels. I always had, and still have to-day, a great doubt as to the inflammatory character of this disease, for I never could notice any great modification in the anatomical structure of the ganglion and vessels, that would be proportioned to the vast extension of the disease over the whole body and for a long time.

I do not believe in the presence of any microbic element in the nourishing fluids, nor in any modification in the chemical or physical elements of the blood, nor in any disturbance in the physiological functions of any organ or system of organs as connected with the primitive origin of this disease, until reliable proofs appear to advance a different opinion.

I must not forget, before closing the diagnosis, to mention the circumstance that frequently local lymphangitis affects the intra-maxillary and inguinal ganglion, and that the first one very often terminates into chronic glanders, if proper care is not taken to effect a speedy cure. The lymphangitis of a posterior leg is always of a more inflammatory type ; generally it terminates well and remains of a benign character. It also happens very often that accidental wounds or wounds consequent to an operation, are followed by ulcerous lymphangitis on their peripheries ; that constitutes local farcy ; it is easily cured, but if neglected, is liable to poison the whole system, and degenerate into general farcy.

In support of my considerations about this lymphatic disease, I will relate several cases of my personal experience :

A heavy truck horse of Geo. Blake & Co., Stockton, working every day up to the evening of October 4, 1884, (having been osing flesh for two weeks before) was found in great distress, having difficulty in moving the hind legs, especially the left one; noticed a hard lump on both crural regions and under the abdomen; various cardeous swellings on tibial region of left leg, covering the external surface only. All these tumors were painful on pressure; complete absence of any tumefied ganglion; visible membranes normal. More lumps appear every day, some very close to one another, on front legs and inside of hind legs; the members of left side are literally covered with buttons, while the right legs have only a few. I counted at the end of the first week about one hundred abscesses; these abscesses that were opened at first cicatrizing quickly, in about two weeks. Horse is moving easier as the abscesses are opened. Fluid evacuated is half coagulated, of an opaque color. The crural muscles are decaying. Not the least tumefaction of lymphatic glands, nor any nasal discharge. Treatment tonic, stimulant and alteratives; horse has a good appetite. The two legs on the left side that were at first covered with abscesses and swollen, became very clean and sound in about five or six weeks, while in the two right limbs affected later a similar change. But cold, rainy weather set in, and the patient falls into a complete marasmus; died January 28, 1885, after an illness of about four months. Eight days before death I noticed a piece of the skin, of the size of a man's hand, fall off, as by dry gangrene, inside the fetlock of left fore-leg. On post-mortem examination I found all viscera sound and clean; only yellow serous infiltration under the skin in those places where swellings existed before death. All organs pale, very little blood in the body. This was certainly not farcy; the horse was always kept on the same premises, no measures of prevention or disinfection were taken, and no case of glanders or farcy appeared on any of the other five horses.

In January, 1886, I found at the ranch of John Ellis Lathrop, three mules affected with this mule-lymphangitis, and one horse suffering from chronic nasal gleet. One mule, bleeding from the nose, was taken out of a plow-team of eight mules. These four animals were running loose in the corrall with forty-eight other mules, feeding in the same manger, and drinking from the same troughs; found blood and mucous discharges on the wagons and fences of the corral; in the three watering troughs I noticed discharged matter floating on the surface and some that went down to the bottom. These animals were taken to another place and killed three or four weeks later. On opening one of them, I found the lungs perfectly sound, and the nasal membrane all rotten. Never had this farmer, to my knowledge, another animal affected with this disease before or after this time. I will mention that if these animals had been glandered, it would be a surprising wonder that the loss at this ranch was so small and the contagious virus so weak and powerless.

John Wagner, of Atlanta, who had lost twelve mules 1883–1884, called on me to examine his stock. In the field I found a horse, working in a team of eight horses, showing sores on both nostrils, some of which were cicatrized; the discharge was mildly abundant, and often mixed with blood; three large hypertrophied glands between the jaws; nasal membrane of rose color. Horse was fat, sleek, in a very good working condition; all other horses and mules sound. This horse was reported two years later to be about in the same condition and working all the time without any danger of contagion to other animals. I never heard that man complain of having lost another horse or mule.

Mr. Heining, of Salida, Stanislaus Co., had one mule taken sick while at work, and in twenty-four hours the nasal passage and larynx were so gravely affected as to cause choking. That mule died in two days. A week later I was called again to his place to attend to the family horse, affected with anemia, and was astonished to find, separated in another stable, two young fat mules, that had both nostrils covered with sores. Mr. Heining received these mules, as a present, from a neighbor, who having lost several animals from the same complaint, had resolved to kill these two. Mr. Heining, being a man of means and of study, wanted to experiment with these mules. So he treated them in most any imaginable manner; they broke out on the body and legs, but improved in the course of a few months, so well that he put them to work, drove around the country with them and mixed them up with his other stock. One of them happened to be overheated and died. The other has recovered entirely, and showed last August, 1888, clean legs, all swellings and tumors of the body had disappeared, only white scars visible on such places where large sores had existed; two inter-maxillary glands still hypertrophied; a light, serous, watery discharge from both nostrils, various cicatrices of old sores visible in both nasal cavities. Mule is lively and in good health. That mule is cured and never had the glanders.

Ed. Hall, of Tarlock, works from sixty to seventy head of stock, and lost several years ago about $2,500 worth of mules within six months. On examining his stock, I found two horses and a mare, with a sucking colt by her side, having a free double discharge of good character and two soft glands loose ; this mare showed the same symptoms at the time the mules were dying, but was not glanderous, as proved not only by the character of her symptoms, but by the soundness of the colt and the other stock. And still the mare and the two horses became affected at the same time and in the same pasture as the dead mules, two years before.

I will now report one more instance in support of the non-cantagiousness of this affection. Three farmers near Modesto had turned their working stock in an alfalfa pasture on the west side of the San Joaquin River, where about four hundred head of stock, horses, mules, brood mares and colts, were kept together about four months. About June 1st they took their animals, one hundred and thirty head in all, home and fed them on grain and hay, to prepare the same for work in the harvest. Hardly three weeks later, each one of these farmers had one mule complaining of this mule disease ; one of David Kerr was all gangrened in the head, and killed after three or four weeks. The mule of James Kenealy broke out in a hind leg ; I lost sight of him ; the third one belonged to W W. Stone, and received my close attention for one month. This mule was three years old and fat ; broke out on legs and body, having, inside of one week, over one hundred abscesses and buttons ; both nasal cavities were gangrened, but there never was any œdema in the glottis. This mule was lively, ate well, and had all the time a splendid coat ; but gradually the disease progressed, and before the farmer concluded to destroy her I was allowed to experiment on her. I inoculated an old mare in three places, one on the left costal region, and one on each side of the neck, by making a cut about two inches long and pouring the fluid extracted from a newly opened abscess of the mule in the pocket of these cuts, and closed them up with one stitch. Then I coated a small sponge over with the nasal discharge of the same mule by holding and turning the sponge around in the nostrils, and introduced this same sponge in both nostrils of the mare, for a few minutes. At the same time, the mare was tied up near the mule, eating out of the same manger and drinking from the same pail. Fourteen days later Mr. Stone resolved to kill his mule, and my mare was taken out and destroyed. The three wounds where I had introduced the matter taken from the mule, were giving a good deal of suppuration of a very healthy character but no swelling, nor cord, nor tumor, could be detected either around the three inoculated wounds nor on any part of the body. The nasal cavities were clear, and no ganglion swollen. The mare was declared sound.

No sick animal could be found on the premises of either one of these three farmers, nor in the pasture. In all certainty these mules were not glandered, unless it be the acute type ; nor was there any contagious virus existing at the home places nor in the pasture. What was the real cause of this disease here? The animals had pure air, good water, good nourishment and no work ; and good, clear weather, without frost or rain, existed at the time.

To prove the change of original lymphangitis into farcy and glanders, I will relate the two following cases :

About four or five years ago a fast roadster, Lightfoot, kept in a very good livery stable in Stockton, was found suddenly, in the morning, bleeding from the right nostril. The right intermaxillary gland was very large and painful, sending a cord toward the right nostril, lip on same side very much swollen ; inside of right nostril a sore three inches long by half an inch wide. This was a lymphangitis of a benign character. In a few weeks the horse recovered, but several months later I condemned him for confirmed chronic glanders, having a hard adherent gland, a bad nasal discharge and two indurated ulcers, all on the *left* side, while the primitive lymphangitis was on the *right* side.

About June, 1888, I examined at the home of Mr. Hamilton, near Salida, a mare which had been sick for several months. I noticed a splendid lustrous coat, the appetite very good, the crural muscles of left hind leg all decayed, same leg swollen below ; from twenty to twenty-five sores on the inside surface of the same leg ; those opening on the lymphatic vessel discharged a yellow fluid half coagulated ; some of them recently opened gave exit to healthy, limpid lymph ; a few of them evacuating a white, cheesy fluid. No swollen glands nor nasal discharge, nor farcy lesions at any other place. Diagnosis : lymphangitis of the inguinal and crural ganglions, but of mixed character. Inside of two weeks the mare showed a great improvement, the most of the sores healed, a few new ones appeared on the mammary gland, the swelling of the leg went down, mare rested better and moves easier. But suddenly a cold north wind set in, causing a chill through the whole system, and twenty-four hours later the hair was staring, coat dry, adherent, appetite bad, expression dull, animal weaker, the affected leg swollen considerably, having the appearance of farcy infiltration ; the aspect of the sores was bad, fluid discharged was white, looks like matter. Mare condemned as affected with farcy and destroyed.

From all the above considerations, theoretical and practical, I am induced to draw the following conclusions :

1st. That this disease affects the lymphatic system, locally or generally.

2d. That it is non-contagious at the beginning, although it might be infectious ; and that where several animals on the same premises are affected, the disease is caused by similar mysterious influences of locality, weather and climate, acting on all the animals in a like manner, without the existence of a contagious principle.

3d. That with the progress of the disease, the animal organism might undergo such modifications as to transform the primitive lymphangitis into a farcinous lymphangitis, and then develop glanders and farcy.

4th. That the disease resists all kinds of treatment and is fatal to all mules, while the majority of horses will partially recover from its effects.

5th. That the presence of a horse affected with chronic glanders in a band of these diseased animals, cannot lead to the conclusion that the disease is of a glandered nature in every case, no matter how suspicious the appearance may be.

*Prognostic.*—Very serious and fatal. Death occurs almost in every case, if the animal is not destroyed by the hands of its owner. Those mules which are affected only on the body and legs, can live six months or a year if properly cared for, although losing strength on account of the great quantity of lymph which is detracted from its natural course and is a direct loss to the general nutrition. When the same disease breaks out in the nasal cavity and larynx, it causes a great difficulty in the act of respiration, and thereby greatly impedes the complete arterization of the blood, besides indirectly impairing the health of the animal, as the inspired air, when going over the gangrened sores, absorbs a diseased, decomposed material, that is introduced through the pulmonary capillaries into the blood and constitutes a permanent danger for blood poisoning.

In horses affected in the same manner, the disease remains stationary and benign for a long time, if they are in a sufficiently good condition ; and many will recover except so far as the lesions in the nasal cavity are concerned, which will constitute a permanent light discharge, render the horse unsound and constantly suspicious. But with horses there is always great danger of this disease turning into glanders, whenever a chill, or a disturbance in the digestion, or a weakness in the constitution through heavy labor, insufficient feeding or exposure to inclement weather, or the consecutive effects of any acute fever, cause a change in the general nutrition and in the functions of the absorbing vessels and the whole lymphatic system. Mules might likewise become glandered from similar changes, but the disease will generally affect a mixed or bastard type, or an acute character.

*Treatment.*—It is my conviction that this disease is incurable in mules, and therefore I would propose, in every case, to destroy all thus affected, for the sake of humanity as well as a precautionary measure against possible glanders and farcy.

If horses are in good condition, they might be submitted to local and general treatment, receive proper care and nursing, pure air and substantial food. Above all they must be kept separated from the other stock. The healthy animals ought to be removed to another locality on high and dry lands if possible. This measure will generally check the further outbreak of the affection.

